# Functional Implications of Neurotransmitter Segregation

**DOI:** 10.3389/fncir.2021.738516

**Published:** 2021-10-13

**Authors:** Fredy Cifuentes, Miguel Angel Morales

**Affiliations:** Departamento de Biología Celular y Fisiología, Instituto de Investigaciones Biomédicas, Universidad Nacional Autónoma de México, Mexico City, Mexico

**Keywords:** cotransmission, co-release, segregation, classical transmitters, cotransmitters, plasticity

## Abstract

Here, we present and discuss the characteristics and properties of neurotransmitter segregation, a subtype of neurotransmitter cotransmission. We review early evidence of segregation and discuss its properties, such as plasticity, while placing special emphasis on its probable functional implications, either in the central nervous system (CNS) or the autonomic nervous system. Neurotransmitter segregation is a process by which neurons separately route transmitters to independent and distant or to neighboring neuronal processes; it is a plastic phenomenon that changes according to synaptic transmission requirements and is regulated by target-derived signals. Distant neurotransmitter segregation in the CNS has been shown to be related to an autocrine/paracrine function of some neurotransmitters. In retinal amacrine cells, segregation of acetylcholine (ACh) and GABA, and glycine and glutamate to neighboring terminals has been related to the regulation of the firing rate of direction-selective ganglion cells. In the rat superior cervical ganglion, segregation of ACh and GABA to neighboring varicosities shows a heterogeneous regional distribution, which is correlated to a similar regional distribution in transmission strength. We propose that greater segregation of ACh and GABA produces less GABAergic inhibition, strengthening ganglionic transmission. Segregation of ACh and GABA varies in different physiopathological conditions; specifically, segregation increases in acute sympathetic hyperactivity that occurs in cold stress, does not vary in chronic hyperactivity that occurs in hypertension, and rises in early ages of normotensive and hypertensive rats. Given this, we propose that variations in the extent of transmitter segregation may contribute to the alteration of neural activity that occurs in some physiopathological conditions and with age.

## Introduction

Neurons have two types of synapses that enable communication between them and their targets: electrical synapses where neurons are connected by clusters of intercellular channels called gap junctions (Bennett and Zukin, 2004), which allow ions and small molecules to flow between cells; and chemical synapses where neurons require a chemical mediator to transmit signals. These mediators, known as neurotransmitters, are synthesized in the neuronal body or in axonal presynaptic terminals, stored in vesicles and released when terminals are depolarized by the arrival of an action potential. Neurotransmitters then diffuse through the space between neurons, bind to specific protein receptors in the neural targets and finally change the membrane potential.

The concept of chemical neurotransmission was proposed by Langley at the beginning of the twentieth century (Langley, 1906), and around the same time the idea of a specialized zone for the transmission of signals between neurons, termed synapses, was introduced by Cajal and Sherrington (see Cowan and Kandel, 2001). Since then, the idea of synaptic transmission has been central to the study and understanding of neuronal communication. Once the existence of chemical synapses and neurotransmitters was clearly demonstrated by Loewi (1921), it was proposed that each neuron uses a single neurotransmitter at all its synapses. The concept of one neuron—one transmitter was termed Dale’s Principle by Eccles based on an interpretation of a concept of Dale (Eccles, 1957) and became accepted in the following decades.

## Neural Cotransmission

In the early 1960s, some findings started to challenge Dale’s Principle; for instance, de Iraldi et al. (1963) demonstrated the presence of more than one neurotransmitter in the synaptic endings of the rat anterior hypothalamus. Owman (1964) suggested that noradrenergic sympathetic nerves in the rat pineal gland also store 5−hydroxytryptamine, thus containing two types of monoamines. In the following decade, it was demonstrated that ATP and norepinephrine (NE), two co-localized neurotransmitters, were functional in smooth muscle of the gut of different species (Burnstock et al., 1970; Burnstock, 1976), and later the occurrence of somatostatin-like immunoreactivity in some sympathetic noradrenergic neurons was shown (Hökfelt et al., 1977). In his pioneering work, Burnstock (1976) coined the concept of cotransmission and classified the participating neurotransmitters as classical and cotransmitter, the former, mostly small molecules, bind ionotropic receptors and evoke fast depolarization or hyperpolarization of the membrane potential, and the latter, usually peptides, bind metabotropic receptors and slowly modulate the action of the classical neurotransmitters. Although there are exceptions to this classification, some substances can serve interchangeable roles as transmitter or modulator, classical transmitters can modulate the action of other transmitters and peptides can serve as classical transmitters (Svensson et al., 2019). In the following years, many cases of cotransmission were demonstrated between classical and cotransmitters in a substantial number of synapses (Morales et al., 1995; Salio et al., 2006; Hökfelt, 2009; Burnstock, 2014; Nusbaum et al., 2017; Svensson et al., 2019). In addition, cotransmission between two or more classical transmitters has been also shown (Jonas et al., 1998; Gutiérrez, 2009; Elinos et al., 2016; Takács et al., 2018; Granger et al., 2020; Ranjbar-Slamloo and Fazlali, 2020). Burnstock argued that cotransmitter composition shows considerable plasticity, including during development and aging, in physiopathological conditions, and following trauma or surgery (Burnstock, 2013).

It was proposed that neuronal cotransmission can be carried out by routing the neurotransmitters to all presynaptic terminals, followed by their co-release (Burnstock, 1990), which gave rise to the updated version of Dale’s Principle stating that a neuron releases the same set of transmitters from all its terminals (Eccles, 1976, 1986). If two classical transmitters were co-localized and co-released this would imply that some neurons release fast excitatory and inhibitory neurotransmitters, and that such concurrent excitation and inhibition to downstream neurons might promote an excitation/inhibition balance in neuronal circuits (Shabel et al., 2014; Saunders et al., 2015). To provoke the specific action of each transmitter, neurons would use specialized mechanisms such as localizing and releasing transmitters from different synaptic vesicles, which may have different release probability, different coupling to presynaptic Ca^2+^ channels and frequency dependence (Kupfermann, 1991; Takács et al., 2018; Silm et al., 2019). As an alternative, transmitters co-localized in the same vesicles might be differentially released by distinct kiss-and-run-like mechanisms (Liang et al., 2017). Even if two classical transmitters were simultaneously released, mechanisms like different diffusion rates or enzymatic degradation would allow neurons to generate temporally and spatially specific signals (de Wit et al., 2009). Another mechanism to produce different and specific signals by each transmitter is by routing them to different distant or neighboring neuronal processes, which allow neurons to localize and release neurotransmitters independently at distinct terminals of single neurons. This modality would allow the targeting of each transmitter to different postsynaptic compartments and the separate modulation of each of them (Tritsch et al., 2016). This type of cotransmission has been termed neurotransmitter spatial segregation or just segregation (Sossin et al., 1990; Hattori et al., 1991; Morales et al., 1995; Sámano et al., 2012; Fortin et al., 2019; von Twickel et al., 2019; Granger et al., 2020) or compartmentalization by others (Galván and Gutiérrez, 2017).

## Neurotransmitter Segregation

Initially, most of the work on cotransmission agreed with the first idea of co-localization and co-release of the same mix of neurotransmitters from all axon endings (Chan-Palay and Palay, 1984; Burnstock, 1990; Boulland et al., 2009; Vaaga et al., 2014; Takács et al., 2018; Aldrich, 2019). However, a significant amount of evidence supporting neurotransmitter segregation, both in invertebrates and mammals, either in the central or peripheral nervous system, has been accumulated in the past few decades (Sossin et al., 1990; Hattori et al., 1991; Morales et al., 1995; Sámano et al., 2006, 2009, 2012; Vaaga et al., 2014; Mingote et al., 2015; Saunders et al., 2015; Lee et al., 2016; Lamotte d’Incamps et al., 2017; Aldrich, 2019; Apergis-Schoute et al., 2019; von Twickel et al., 2019; Granger et al., 2020). Like cotransmission by co-localization, neurotransmitter segregation may occur between classical transmitters and cotransmitters, and between two classical transmitters.

Neurotransmitter segregation can be achieved in two ways, either by sorting neurotransmitters to distant processes that innervate separate targets, or by sorting them to neighboring axonal boutons, raising the possibility that some of these boutons contain different neurotransmitters and face close or even the same targets. The first type of segregation was shown in *Aplysia californica* by Fisher et al. (1988) and Sossin et al. (1990). They demonstrated that in the bag cell soma, two neuropeptides derived from the same prohormone are stored in distinct vesicles, and processed and targeted to separate neuronal processes or distant process (Fisher et al., 1988; Sossin et al., 1990). An example of segregation of the transmitters glutamate (Glu) and dopamine (DA), was shown in the neurons of the substantia nigra pars compacta/ventral tegmental area (SNc/VTA) of the lamprey that target separately the striatum and the optic tectum (von Twickel et al., 2019). However, segregation to neighboring endings has been the most common type of segregation demonstrated to date (Hattori et al., 1991; Morales et al., 1995; Sámano et al., 2006, 2009; Mingote et al., 2015; Saunders et al., 2015; Zhang et al., 2015; Fortin et al., 2019; Granger et al., 2020). Hattori et al. (1991) described that in the rat striatum, a single nigrostriatal dopaminergic neuron gives rise to two types of *en passant* synapses, asymmetric and symmetric, and that dopaminergic transmitter markers occur only in the symmetric synapses.

Based on the work of Morales et al. (1995), which suggested segregation of acetylcholine (ACh) and met-enkephalin (m-Enk) in the rat superior cervical ganglion (SCG), we have confirmed the existence of neurotransmitter segregation to neighboring boutons in axonal terminals of sympathetic preganglionic neurons (SPN) of cat and rat SCG (Sámano et al., 2006, 2009). These neurons have their cell bodies in the spinal cord and send their axons throughout spinal nerves to the sympathetic ganglia. We have found that cell bodies of SPN containing peptides, invariably also contain the classical preganglionic sympathetic transmitter ACh, while in their axonal varicosities some of them express separately cholinergic markers and peptides (Sámano et al., 2006, 2009). We confirmed the preganglionic origin of the varicosities localizing peptides and lacking ACh, since they faded after ganglia denervation (Sámano et al., 2006). Later, we demonstrated that this type of segregation occurs between two classical neurotransmitters, ACh and GABA, which exert excitatory and inhibitory effects on ganglionic neurons (Elinos et al., 2016). Likewise, in cultured sympathetic ganglionic neurons, we found segregation of two other classical transmitters, ACh and NE (Vega et al., 2010).

Segregation of different neurotransmitters has been demonstrated in various regions of the central nervous system (CNS), for example, Saunders et al. (2015) found that *globus pallidus externous* neurons projecting to the frontal cortex segregate ACh and GABA to different presynaptic terminals, which face distinct excitatory or inhibitory postsynaptic sites, while they also detected terminals storing ACh and GABA in different vesicles of the same terminal (Saunders et al., 2015). They also found that cortical vasoactive intestinal peptide (VIP)^+^/choline acetyltransferase (ChAT)^+^ interneurons that co-release GABA tailor (segregate) their transmitters to different terminals of the same axons depending on the target neurons (Granger et al., 2020). Some axons of dopaminergic/glutamatergic neurons of the mouse VTA spatially segregate DA and Glu to different terminals, since some Glu^+^ boutons do not express tyrosine hydroxylase (TH; Mingote et al., 2015). One of the clearest pieces of evidence of neurotransmitter segregation comes from Zhang et al. (2015) who showed ultrastructural evidence of different release sites of DA and Glu within a single axon of VTA neurons. Segregation of Glu and GABA in mossy fibers (MF) of the hippocampus has been demonstrated by Galván and Gutiérrez (2017). They showed that MF co-localize Glu and GABA and sort them to distant terminals innervating interneurons of different strata of hippocampus, they referred to this process as compartmentalization (Galván and Gutiérrez, 2017). Another well-documented case of neurotransmitter segregation occurs in retinal cholinergic amacrine cells, which contain and release ACh and GABA (Duarte et al., 1999; Lee et al., 2010; Hanson et al., 2019; Pottackal et al., 2020). It has been also shown that glycine (Gly) and Glu are segregated in vesicle glutamate transporter 3 (VGluT3^+^) amacrine cells (GAC) in baboon and mouse retinas (Marshak et al., 2015; Lee et al., 2016, respectively).

## Neurotransmitter Segregation Plasticity

**FIGURE 1:**
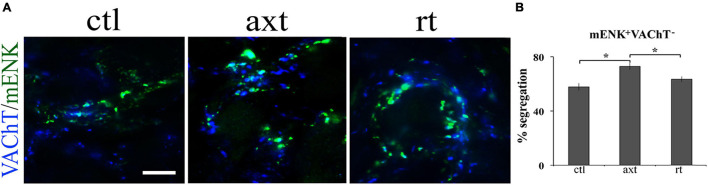
Axotomy increases the segregation of VAChT from m-Enk-containing varicosities in the SCG. Exogenous NGF prevents this increase. **(A)** Micrographs showing SCG sections double-immunostained for VAChT (blue) and m-Enk (green) in control (ctl), after axotomy (axt) and after axotomy and NGF restitution (rt). **(B)** Bar graph showing the degree of segregation in the three experimental conditions. Axotomy significantly increased the segregation of VAChT from m-Enk-containing varicosities, while after axotomy and NGF restitution the segregation of VAChT from m-Enk was significantly different from the axotomy group, but not from control, indicating that exogenous NGF prevents this increase. *p < 0.05; calibration bar 10 μm (reproduced with permission from Vega et al., 2016).

**FIGURE 2:**
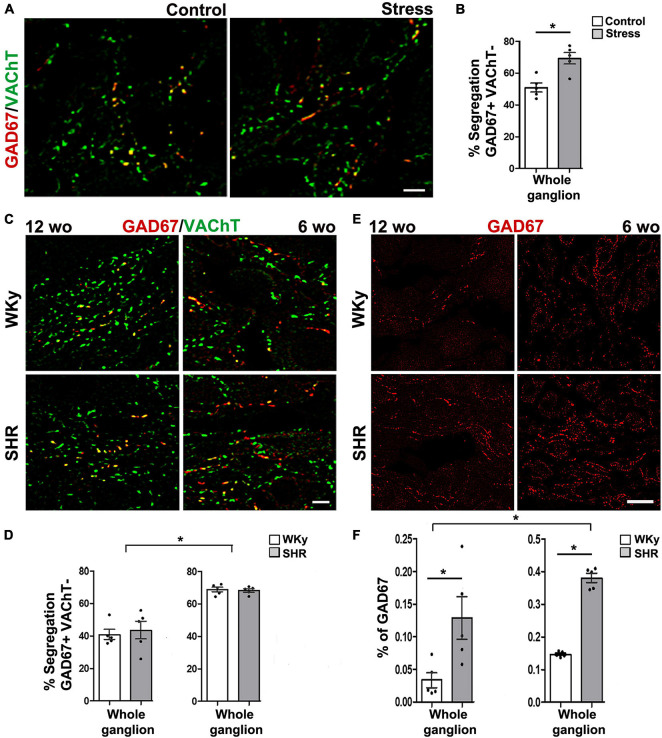
Segregation of acetylcholine (ACh) and GABA varies in different physiopathological conditions. **(A)** Merged images showing the immunolabeling of GAD67 (marker for GABA; red), vesicular ACh transporter (VAChT; marker for ACh; green), and co-localization of both labels (yellow) in superior cervical ganglion (SCG) from control and cold-stressed rats. **(B)** Bar graph showing that cold stress increased the percentage of segregation of VAChT/GAD67 compared with control rats. Scale bar = 10 μm, *p < 0.05. **(C)** Merged images showing the co-localization (yellow) of GAD67 (red) and VAChT (green) in SCG from 12-week-old (wo; left panels) and 6-wo (right panels) SHR and WKy rats. **(D)** Bar graphs showing that segregation is greater in the SCG of 6-wo than in the 12-wo of both strains of rats; segregation is similar between ganglia from SHR and WKy rats. **(E)** Immunostaining for GAD67 in SCG from SHR and WKy rats at 12-wo and 6-wo. **(F)** Bar graphs showing that the percentage of GAD67-containing varicose fibers increases significantly in SHR at 6-wo and 12-wo, and that it is larger in 6-wo than in 12-wo of both strains. Scale bar = 10 μm (modified from Merino-Jiménez et al., 2018).

One remarkable characteristic of neurotransmitter segregation is its plasticity, since it changes under different experimental conditions, as well as in some physiopathological conditions or with age (Merino-Jiménez et al., 2018). We have demonstrated that the pattern of segregation is not static and invariable; rather, it is a plastic property that changes depending on neuronal requirements. For instance, we found that, in cultured ganglionic neurons, exogenous neurotrophic factors modify segregation between NE and neuropeptide Y, and between NE and ACh (Vega et al., 2010). Segregation between ACh and m-Enk can be modified by endogenous neurotrophic factors in the SPN *in vivo.* We showed that axotomy produces a decrease in endogenous nerve growth factor (NGF) content and an enhancement of ACh and m-Enk segregation in the rat SCG; these effects were counteracted by NGF administration (Vega et al., 2016; Figure 1). Considering these findings, we wondered whether neurotransmitter segregation varies in different physiopathological conditions, such as stress and hypertension. To answer this question, we explored the expression and segregation of ACh and GABA in the rat SCG under stress condition (induced by cold) and in a rat model of hypertension, spontaneously hypertensive rats (SHR). We found that the degree of segregation of these two neurotransmitters increases in the acute sympathetic hyperactivity that occurs in cold stress, but not in hypertension that courses with chronic sympathetic hyperactivity. However, in hypertension there is an increase in GABA expression (Merino-Jiménez et al., 2018; Figure 2). Likewise, a remarkable study by Fortin et al. (2019) demonstrated neurotransmitter segregation plasticity in the dopaminergic-glutamatergic neurons of the VTA, which innervate striatal neurons. They found that in these dual phenotypical neurons, segregation of DA and Glu is regulated by target-derived signals. They suggested that VTA axonal terminals release either DA or Glu, depending on the interaction with ventral striatal neurons (Fortin et al., 2019). Similarly, Galván and Gutiérrez (2017) showed that in MF of the hippocampus, Glu and GABA are compartmentalized (segregated) and released (independently or jointly) from a single pathway onto different interneuron sets, in a target-dependent manner.

## Does Transmitter Segregation Result in Improved Neuronal Function?

As discussed above, classical neurotransmitters and cotransmitters released from the same axon terminal can act concurrently on the same target, whereas if they are segregated into different neuronal processes, once released, they can act separately from each other. One improvement attributable to neurotransmitter segregation thus might be that the neuronal signaling repertoire increases. In their initial work, Fisher et al. (1988) and Sossin et al. (1990) demonstrated that bag cell peptides (BCP) and egg-laying hormone (ELH) showed two spatially segregated functions, namely, BCP exert an autocrine excitatory function on the bag cells, while ELH exerts a paracrine action on nearby neurons and peripheral tissues. The authors suggested that by segregating their transmitters, neurons can expand the array of mechanisms used to generate or to improve specific interactions between neurons, allowing them to modify specific presynaptic terminals during plastic neuronal experiences (Sossin et al., 1990). In relation to the segregation of DA and Glu in dopaminergic neurons of the lamprey, these SNc/VTA neurons in addition to co-release of DA and Glu into microdomains of the striatum, can also differentially influence diverse brain areas by segregating DA and Glu. Thus, the co-release of Glu and DA may be advantageous in the striatum, whereas the action of Glu release alone could modulate processes in the motor center (tectum) that controls eye and orienting movements (von Twickel et al., 2019).

Regarding segregation of neurotransmitters within synaptic endings of the same axon, several research groups have proposed diverse correlations between segregation and function. Hattori et al. (1991) were the first to propose the presence of neurotransmitter segregation within synaptic endings of the same neuron. They proposed that this putative ability of segregated neurotransmitters “*may enhance the computational processing of the nervous system, and may provide new insights into the functional significance of the well-documented multiple coexistence of neurotransmitters in single neurons*” (Hattori et al., 1991). In a similar way, it has been postulated that by segregating ACh and GABA to different terminals, retinal amacrine cells can regulate the firing rate of direction selective-ganglion cells (DSGCs) in response to a bar of light moving in different directions across the retina (Duarte et al., 1999). Movement in the preferred direction activates release of ACh, which fires a series of action potentials in the DSGCs, whereas movement in the opposite null direction releases GABA that evokes little or no response (Duarte et al., 1999). Other authors found that starburst amacrine cells (SAC) make cholinergic synaptic contacts on DSGC that fire when light comes from all directions to detect image motion, but SAC make GABAergic synapses on DSGCs, which fire only when light comes from the null direction, allowing the detection of image motion direction (Lee et al., 2010). More recently, it was shown that differential connectivity of SAC GABAergic and cholinergic signals on the DSGC, produces excitation/inhibition timing differences that generate direction selectivity (DS; Hanson et al., 2019). Pottackal et al. (2020) have proposed that in retina, DS represents an elementary sensory computation that can be related to underlying synaptic mechanisms of the DSGC that respond strongly to visual motion in a “preferred” direction and weakly to motion in the opposite, “null” direction. They showed that the preferred direction-untuned excitation is related to cholinergic signals, while the null direction-tuned inhibition depends on GABAergic input. Similarly, segregation of Gly and Glu has been shown in GAC. These cells form glycinergic synapses with uniformity detectors (or suppressed-by-contrast cells), while they release Glu to excite mainly OFF alpha ganglion cells. This coordinated inhibition and excitation of two separate neuronal circuits by a single interneuron is used to facilitate differential detection of visual field uniformity and contrast (Lee et al., 2016).

The compartmentalization of Glu and GABA by the MF in the hippocampus of juvenile rats enables different actions onto interneurons, which depending on the hippocampal strata where are located, they either receive both glutamatergic and GABAergic signals, or exclusively a glutamatergic signal (Galván and Gutiérrez, 2017). Interestingly, these authors found a correlation between this Glu and GABA compartmentalization and synaptic plasticity. Glutamatergic/GABAergic MF contacting the stratum lucidum interneurons undergo glutamatergic long-term depression (LTD), and GABAergic long-term potentiation (LTP), while interneurons located in stratum radiatum despite receiving this dual signaling, do not display such plasticity, accordingly they propose a counterbalanced compensatory plasticity of two neurotransmitters released by different terminals of the same pathway (Galván and Gutiérrez, 2017). Another study demonstrating a similar target-specific co-release of neurotransmitters, ACh and GABA, in ChAT^+^ interneurons of mouse was recently communicated in a comprehensive article by Granger et al. (2020). They found that these ChAT^+^ interneurons are positive to VIP and present two distinct populations of pre-synaptic terminals: a subset that can release both GABA and ACh and another subset that can release only GABA (Granger et al., 2020). The segregation and independent release of ACh and GABA from different presynaptic terminals suggests a coherent model of the net effect that these neurons have on cortical circuits: ACh-mediated excitation of other VIP^+^/ChAT^+^ disinhibitory interneurons, while GABA provokes disinhibition on other neurons, with the combination of these two ACh- and GABA-mediated actions provide a powerful activating signal to local cortical areas (Granger et al., 2020). A functional correlation of DA segregation in VTA neurons has been proposed by Morales and Margolis (2017), who have been interested in learning about the function of these molecularly diverse neurons, which may release DA, Glu or GABA and have central roles in reward-related and goal-directed behaviors. Because of the segregation arrangement of Glu and DA into distinct ultrastructural domains of release, mesoaccumbens fibres of VGLUT2^+^/TH^+^ neurons from the VTA can exert two separate effects on the nAcc: one postsynaptic Glu-excitatory over nAcc neurons and another presynaptic D2 receptor-mediated in mesoaccumbens fibres that modulate Glu release. In this way the segregation of DA from Glu allows VTA neurons to regulate the amount of excitation of nAcc neurons (Zhang et al., 2015). In other regions, such as the olfactory bulb, release of DA and GABA with distinct spatiotemporal profiles in TH-positive neurons in the glomeruli may allow these cells to control different synaptic timing in intraglomerular and interglomerular circuits (Borisovska et al., 2013).

**FIGURE 3:**
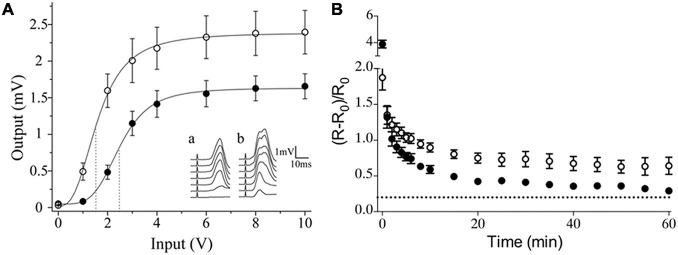
Sympathetic ganglion neurons exhibit different activation and expression of long-term potentiation (LTP) according to their intraganglionic regional location. **(A)** Input-output curve of ganglionic transmission recorded in the external carotid nerve (ECN; ∘) and the internal carotid nerve (ICN; •), which contain axons of caudal and rostral neurons, respectively. Stimuli of similar amplitude evoked a greater response in the ECN than in the ICN. Insets show a set of compound action potentials (CAPs) evoked by each input intensity tested, recorded in the ICN (a) and in the ECN (b). **(B)** Time course of ganglionic LTP showed as ΔR/R0 (mean ± SEM), evoked in the caudal region, recorded in the ECN (∘) and in rostral region, recorded in ECN (•) (reproduced with permission from Elinos et al., 2016 and Martínez et al., 2020).

**FIGURE 4:**
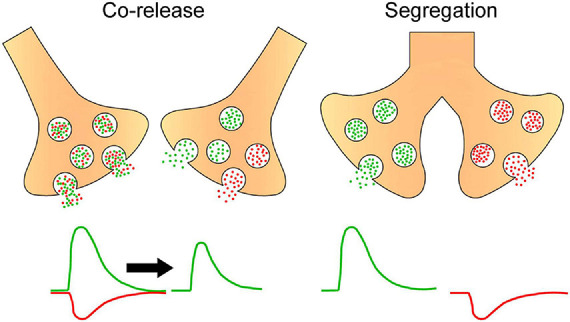
Hypothetical scheme postulating that segregation and independent release of ACh and GABA result in reduced GABA inhibition of cholinergic effects. Drawing depicts presynaptic boutons either co-releasing ACh (green circles) and GABA (red circles) from the same or different vesicles or releasing these neurotransmitters independently from separate endings. In the first case, the excitatory action of ACh coincides with the inhibitory action of GABA, resulting in a reduced cholinergic effect, while in the independent release each transmitter exerts its effects separately, avoiding the inhibitory action of GABA on the effect of ACh.

Like in other neuronal structures, neurotransmitter segregation in sympathetic ganglia could influence synaptic transmission. We showed that segregation of ACh and GABA in the SPN axon endings is not homogeneously distributed across different regions of the rat SCG. Rather, the segregation level is higher in the caudal than in the rostral region (Elinos et al., 2016), and this greater segregation in the caudal region is correlated with stronger synaptic transmission and enhanced synaptic plasticity as evidenced by a greater expression of ganglionic LTP (Elinos et al., 2016; Martínez et al., 2020; Figure 3). We propose that the co-release of ACh and GABA from single boutons making synapse onto the same postganglionic neuron allows GABA to inhibit ACh transmission, whereas the release of only GABA to distinct postganglionic neurons produces less inhibition of cholinergic transmission The presence of a larger number of more excitable neurons with a low threshold in caudal region (Li and Horn, 2006) would be consistent with less GABA innervation on these neurons. Accordingly, a functional implication of segregation of ACh and GABA in sympathetic ganglionic synapses could be the modulation of GABAergic inhibition, and thus a larger segregation of ACh and GABA results in less GABAergic inhibition, which may explain the stronger synaptic transmission and enhanced plasticity of caudal region of SCG (Figure 4). We recognize that the difference in compound action potential (CAP) amplitude shown in Figure 3A can be also due to a larger number of caudal than rostral neurons, or the result of a larger number and different type of preganglionic inputs making synaptic contacts on caudal than on rostral neurons. Gibbins and Morris (2006) established that variation in number and type of preganglionic input making synaptic contacts on ganglionic neurons determine the synaptic strength in autonomic ganglia. An equivalent study reproducing the approach of these authors determining the number and type of preganglionic inputs in caudal vs. rostral neurons would help to clarify this issue.

We explored the degree of ACh and GABA segregation in sympathetic ganglia of the rat in the physiopathological conditions of cold stress and hypertension. We found that in stressed rats, segregation of ACh and GABA increases, whereas it did not change in hypertensive rats (Merino-Jiménez et al., 2018). These findings suggest that the acute sympathetic overactivity underlying stress is due to reduced GABA inhibition resulting from the increase in ACh and GABA segregation. On the other hand, segregation level did not vary in hypertension probably because in this pathology there is a chronic increase in sympathetic function, which does not involve GABAergic modulation of sympathetic ganglionic transmission.

To determine whether the degree of segregation varies at different ages we explored ACh and GABA segregation in young, 6-week-old, and adult, 12-week-old, SHR, and normotensive Wistar Kyoto (WKy) rats. In both strains we found larger segregation at the early age (Merino-Jiménez et al., 2018; Figure 2), with segregation decreasing in the older animals. This higher segregation at early age implies independent release of GABA, which probably does not affect ACh actions. Rather, this GABA might exert actions related to development of ganglia, as suggested by others (Wolff et al., 1987). By regulating segregation, GABA released from SPN axon endings can then exert different actions, such as modulating development at an early age, or synaptic transmission as animals become older.

## Conclusion and Future Directions

In the process of cotransmission, neurons can route the same combination of transmitters to all their presynaptic terminals, or segregate and sort the transmitters to separate endings, with the latter capability termed transmitter segregation. It has been demonstrated that segregation shows plasticity as it can be modified according to required synaptic transmission conditions. Here, we reviewed the findings of other researchers and of our own that segregation expands the neural signaling repertory. For example, by means of segregation of its transmitters to separate cell processes, neurons can exert different synaptic actions with distinct transmitters released from separate axon boutons. We present our latest finding showing a functional correlation between the level of ACh and GABA segregation and the strength of synaptic transmission in the rat SCG. Finally, we propose that changes in the degree of transmitter segregation can serve as a basis of variation in neural sympathetic activity that occurs with age and in some physiopathological processes.

Functional connectivity in a neural circuit is determined by the strength, incidence, and neurotransmitter nature of its connections (Chuhma, 2015). Using optogenetics and viral strategies the functional synaptic connections between an identified population of neurons and defined postsynaptic target neurons may be measured systematically to determine the functional connectome of that identified population (Rajendran et al., 2019). In the case of the SCG, the use of these strategies would be relevant to explore whether preganglionic terminals containing ACh and GABA alone or in combination synapse on postganglionic neurons with different phenotype and function.

## Author Contributions

MAM conceived the original idea with the support of FC. Both authors explored related literature and selected the appropriate antecedents, wrote the manuscript, and contributed equal to the final version of the manuscript.

## Conflict of Interest

The authors declare that the research was conducted in the absence of any commercial or financial relationships that could be construed as a potential conflict of interest.

## Publisher’s Note

All claims expressed in this article are solely those of the authors and do not necessarily represent those of their affiliated organizations, or those of the publisher, the editors and the reviewers. Any product that may be evaluated in this article, or claim that may be made by its manufacturer, is not guaranteed or endorsed by the publisher.
